# Retinal Origins of Circadian Photoregulation's Specialized Dynamic Range and Temporal Integration

**DOI:** 10.64898/2025.12.30.695276

**Published:** 2025-12-30

**Authors:** Elliott S. Milner, Hannah A. Blume, S. Navid Mousavi, Michael C. Brown, Franklin Caval-Holme, Nguyen-Minh Viet, Takeharu Nagai, Michael Tri H. Do

**Affiliations:** 1 F. M. Kirby Neurobiology Center and Department of Neurology, Boston Children’s Hospital and Harvard Medical School.; 2 Sainsbury Wellcome Centre for Neural Circuits and Behaviour, University College London; 3 Graduate School of Frontier Biosciences, The University of Osaka

## Abstract

The circadian clock allows organisms to anticipate environmental changes that are driven by the earth’s rotation, and synchronizes with this cycle using light^1^. The clock extracts relevant parameters of illumination, responding selectively to intensities at twilight and above, and to the number of photons counted over an extended time window^2-8^. These hallmark properties are thought to manifest in the brain^9^. Here, we report that they emerge within specific neurons of the eye. These M1 intrinsically photosensitive retinal ganglion cells (ipRGCs) detect light directly, using a receptor called melanopsin, and indirectly, through circuits driven by rod and cone photoreceptors^10^. We find that the balance of intrinsic and extrinsic drives varies widely across M1s, and the population accounts for the dynamic range of circadian photoregulation. Both singly and collectively, M1s approach the level of temporal integration observed behaviorally. The cellular and behavioral levels match when the pupillary light reflex—another M1-driven function—shapes the light that M1s receive. This work reveals how the first steps of sensory processing are precisely formatted for specific tasks. It also introduces bioluminescence-based methods for identifying photosensitive neurons without desensitization, allowing their mechanistic study under physiological conditions.

While visual perception begins when each rod photoreceptor is activated by one photon every ~8 minutes, the circadian clock requires ~70 such activations per second to respond^2,11^. This high threshold allows animals to see in moonlight without shifting their clocks. Moreover, while perception resolves timing in the tens of milliseconds, the clock ignores these dynamics; remarkably, the clock responds similarly to a given photon count whether it is received over milliseconds or minutes, steadily or in pulses^3,4^. This high temporal integration smooths away irrelevant details in the visual scene to favor the representation of overall illumination (irradiance), which reflects the sun’s position in the sky. These distinctions between perception and circadian photoregulation were early hints of mammalian photoreceptors that are not rods and cones. These ipRGCs sense light intrinsically, using the melanopsin photopigment, and extrinsically, by receiving synaptic input from rod- and cone-driven circuits. IpRGCs, especially those of the M1 type, are essential for circadian regulation^12-15^. How M1s support the clock’s high threshold and temporal integration is unclear.

This question has remained elusive for two major reasons. One is that genetically-identified cell types are customarily identified using fluorescence, but even 2-photon imaging produces an estimated 10^4^ photoactivated rhodopsin molecules per rod each second (R*/rod/s), which is a hundred-fold higher than circadian threshold and desensitizes the retina^2,16^. *Ex vivo,* where experimental access is highest, most of this desensitization is irreversible^17^. The second reason is that M1s are not often situated in conceptual frameworks used to study circadian photoregulation^3,4^. Thus, although these neurons have a central role in this process, how they fulfill it is unclear. Studying this topic provides insight into how neural signals are configured for circadian photoregulation and addresses the broad question of how cell types are suited to their tasks.

In the following sections, we will introduce a method to identify M1s with minimal activation, define how synaptic and intrinsic drives combine in these cells at threshold and above, compare M1 thresholds with that of circadian photoentrainment, estimate the level of temporal integration by M1s, and determine how it matches that observed behaviorally.

## Genetic identification of cells with minimal photoexcitation

We tested a bioluminescent reporter, nano-lantern (NL)^18^, for identifying retinal neurons without desensitization. NL is genetically encodable, uses coelenterazine-h (CH) as a substrate, and emits intensely enough for real-time visualization of live cells. We produced a transgenic mouse line carrying Cre-dependent NL (*ROSA26*^*FLEX-NL*^; Supplementary Figure 1) and crossed it with a line expressing Cre from the melanopsin gene locus (Opn4^Cre^)^19^. We provided CH transiently during retinal dissection, so bioluminescence faded with time during experimentation. Cells of varied morphology were bioluminescent, as expected from ipRGC heterogeneity (M1-M6 types; Supplementary Figure 2)^20^.

To ask if bioluminescence drives desensitization, we examined ipRGCs of the M4 type. M4s allow control experiments because they are identifiable without a reporter, and provide a conservative test case because they are bioluminescent in our conditions and no other RGCs are more sensitive^21,22^. We used loose-patch electrophysiological recordings to measure spikes with minimal perturbation (35 °C). We found that control M4s, having NL or CH but not both, fired spikes at low rates that were stable over time (Supplementary Table 1 and Supplementary Figure 2). These M4s had flash-driven thresholds that were comparable to M4s that lacked NL and CH, suggesting that these molecules do not affect physiology^21,22^. Test M4s (NL^+^CH^+^) had baseline firing rates that were elevated at early time points but still lower than those of M4s identified by 2-photon fluorescence^23^. These firing rates diminished across later time points, likely due to the decay of bioluminescence. Despite this elevated baseline firing, bioluminescence-identified M4s had flash-driven thresholds that were similar to those of controls across all time points (−1.04 ± 0.31 log R*/rod, 12 cells; Supplementary Table 1). Step-driven thresholds were comparable to flash-driven thresholds (11 cells, Supplementary Figure 2B). NL appears to be an innocuous reporter even for the most photosensitive outputs of the retina.

## Synaptic and intrinsic photoresponses of M1 ipRGCs

**Figure 1.**
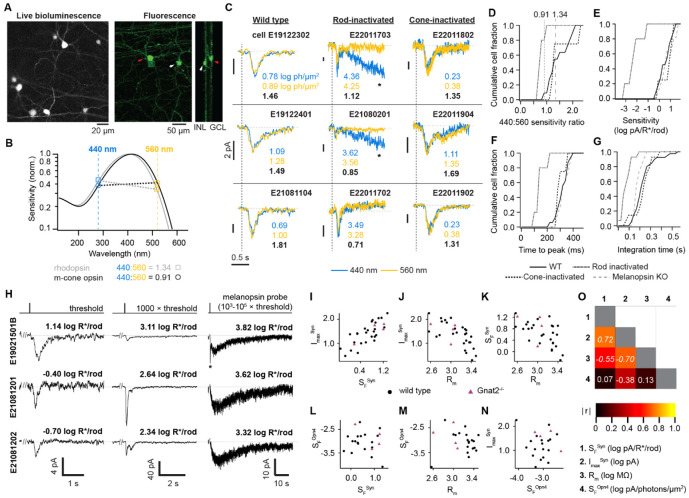
Synaptic and intrinsic drives of M1 ipRGCs **A.** IpRGCs retrograde-transduced with nano-lantern from the suprachiasmatic nucleus. *Left,* live, bioluminescence visualization. *Right,* Projections of a confocal volume through fixed tissue, showing two cells with M1 morphology: one whose soma is in the Ganglion Cell Layer (right, adjacent to labeled axons; red arrowhead) and one in the Inner Nuclear Layer (left, adjacent to dendrites, white arrowhead). **B**. Spectral sensitivities of rhodopsin (grey) and medium-wavelength-sensitive (M) cone photopigment (black). Their sensitivities to 440- and 560-nm light (dashed vertical lines) have ratios of 1.34 and 0.91, respectively. **C**. Example responses to 440-nm (blue) and 560-nm (gold) flashes of wild-type (left column), *Gnat1*^*−/−*^ (center), and *Gnat2*^*−/−*^ (right) M1s. Horizontal dashed lines mark 0 pA; vertical dashed lines mark the onset of a 30-ms flash of 440 or 560 nm. Responses with closely matched amplitudes between the wavelengths were analyzed. Noted are the flash intensities (log photons/μm^2^) and 440:560 sensitivity ratio (black). In *Gnat1*^*−/*−^ retinas, 440-nm flashes needed to be more intense and thus sometimes evoked melanopsin responses (asterisks), which returned to baseline within ~60-70 seconds. Traces are averages of >10 sweeps (500-Hz low-pass filtered for display). −80 mV, 23° C for stability. **D-G**. Cumulative distributions of 440:560 sensitivity ratio (**D**), absolute flash sensitivity (**E**), time to peak (**F**), and integration time (**G**; normalized integral of the response^25,33^) across genotypes (11, 5, 4, and 11 cells from wild-type, *Gnat1*^*−/−*^, *Gnat2*^*−/−*^, and *Opn*^*Cre/Cre−*^ animals; no *Opn4*^*Cre/Cre*^ in **D**). Dashed and dotted vertical lines in **D** mark the expected spectral sensitivity ratio for rhodopsin and M cone photopigment, respectively. **H**. Sample responses from three M1s (rows) to a near-threshold flash (30 ms; left column, average of >10 sweeps); a 1000x brighter flash, which revealed large variation in response gain and kinetics (30 ms; center column, average of 1-3 sweeps); and a flash bright enough to produce a melanopsin dim-flash response (30-200 ms; right column, single sweep). −80 mV, 500-Hz low-pass filtered for display. Horizontal dotted lines (gray) mark 0 pA on each y-axis. Flash intensities are given (R*/rod). Melanopsin responses were generally measured in antagonists of synaptic transmission; in some cases (e.g., **E19021501B**), intrinsic and extrinsic (asterisk) responses were separable without them^24,28,29^. **I-N**. Plots of four response parameters (S_F_^Syn^, synaptic flash threshold sensitivity; I_max_^Syn^, synaptic response amplitude at 1000x threshold; R_m_, membrane resistance; and S_F_^Opn^, melanopsin dim flash sensitivity) measured in single M1s. Some parameters covaried (**I-K**) and others did not (**L-N**). Wild-type (black circles, 30 cells) and *Gnat2*^*−/−*^ M1s (magenta triangles, 4 cells) are plotted together for comparison. **O**. Correlation coefficients for the four parameters (data from wild type only). Spearman r values that were statistically significant are italicized (p ≤ 0.0167 with Bonferroni correction for multiple comparisons).

To enrich for M1 ipRGCs that directly influence the clock, we generated NL-encoding viruses and made injections into the hypothalamus, centered on the suprachiasmatic nucleus (SCN, the central clock, which receives the majority of its retinal innervation from M1s; Figure 1A)^15,24^. We made loose-patch recordings from M1s and found baseline firing rates of 2.8 ± 3.3 Hz (32 cells, 35 °C, Supplementary Figure 2G), similar to those measured previously^23,25^. To our knowledge, the threshold intensity for synaptically-driven M1 spiking has not been precisely defined. We found that flashes evoked firing at different intensities across cells. The mean was 0.50 ± 0.73 log R*/rod (Supplementary Figure 2F) and many cells fired substantially only at >1 log R*/rod. These thresholds were more varied than in M4s (standard deviations of 0.73 and 0.31 log R*/rod, respectively), consistent with M1 heterogeneity^24,26,27^. Most M1s spiked briefly at threshold, indicating drive by synaptic input rather than melanopsin phototransduction (which is much slower)^24,28,29^. The most sensitive M1 light responses appear to be driven by retinal circuits of low sensitivity.

To determine the origin of M1 threshold responses, we compared their spectral sensitivities to those of the retinal photopigments. We made perforated-patch, voltage-clamp recordings and gave near-threshold flashes of 440 and 560 nm (at 23 °C for additional stability). The 440:560 sensitivity ratio should be 1.34 for rhodopsin (peak wavelength sensitivity, $\lambda_{\max}$, of 502 nm) and 0.90 for medium-wavelength sensitive (M) cone photopigment (508 nm; Figure 1B); short-wavelength sensitive (S) cone pigment (358 nm) absorbs these wavelengths poorly (10^−3^ and 10^−7^ of maximum, respectively) and should be negligible at the intensities tested^30,31^. Any melanopsin activation (471 nm for the ground state, the only one detectable after dark adaptation^32,33^) should be clear, with a 440:560 nm ratio of ~20 and distinctly prolonged kinetics^24,25,33,34^. We adjusted the intensities of 440- and 560-nm flashes to obtain responses of similar, small amplitudes (0.04-1.5 log photons μm^−2^, 2-10 pA)(Figure 1C). We divided the response amplitude by the flash intensity to obtain sensitivity^25,35^. The 440:560 nm sensitivity ratio was 1.52 ± 0.42 (11 cells), indicating rhodopsin drive (Figure 1D). The ratio was indistinguishable with cone inactivation^36^ (1.56 ± 0.51, 4 cells, p = 0.95). With rod inactivation^37^, the ratio was 0.84 ± 0.12 (5 cells), consistent with M cone photopigment (p<0.001 and 0.016 for wild-type and cone inactivation, respectively). Furthermore, with rod signaling intact, threshold responses were more sensitive and prolonged (Figure 1E-G and Supplementary Table 2). We also examined melanopsin-null cells and found that they resembled wild-type cells in the integration time of threshold responses and flash sensitivity (Supplementary Table 2). Therefore, the most sensitive photocurrents in M1s are of rod origin.

Next, we asked how synaptic and intrinsic drives combine in M1s. In perforated-patch recordings, we delivered flashes (500 nm) that evoked threshold responses (0.034 ± 0.42 log R*/rod, 4.4 ± 2.0 pA, 27 cells; Figure 1H and Supplementary Figure 3). All cells exhibited the fast response kinetics expected of synaptic drive (247 ± 79 ms integration time, 23 °C). We then delivered more intense flashes, at 1000× threshold to evoke larger synaptic photocurrents and up to 4.95 log R*/rod as needed to evoke melanopsin photocurrent (>40 s decay to baseline)^24,25,28,29,33^. Occasionally, synaptic and melanopsin response phases were well separated, and we measured the peak of the latter directly. Otherwise, we added antagonists of synaptic transmission to isolate the melanopsin response. We obtained peak melanopsin responses of 2-15 pA, which were likely in the linear range and thus readily analyzed^24,25^. We divided the peak amplitude of each response phase by the flash intensity to yield the sensitivity of synaptic (S_F_^Syn^) and melanopsin (S_F_^Opn4^) drives.

We give these anayses in Figure 1I-O (26 cells). S_F_^Syn^ was 0.57 ± 0.49 log pA/R*/rod. Higher S_F_^Syn^ was associated with lower membrane resistance (R_m_) and larger synaptic photocurrent at 1000× threshold (I_1000×_, which was also correlated with R_m_). Notably, M1s with similar threshold sensitivity (S_F_^Syn^) may receive differing amounts of synaptic drive in brighter light, as I_1000×_ responses ranged from 2 to nearly 200 pA. Turning to intrinsic photosensitivity, we found that S_F_^Opn4^ was −3.07± 0.44 log pA/photons/μm^2^ (expressed at 471 nm, the $\lambda_{\max}$ of melanopsin’s ground state, and equivalent to −3.14 log pA/R*/rod). This is orders of magnitude lower than S_F_^Syn^ (p = 0.16). The distributions of S_F_^Opn4^ and S_F_^Syn^ did not overlap, and S_F_^Opn4^ did not show correlations with any measured parameters. To summarize, we observed orderly relationships for synaptic but not melanopsin drives, raising the question of how they combine to shape ipRGC outputs.

## Intensity encoding with synaptic and intrinsic photoresponses

**Figure 2.**
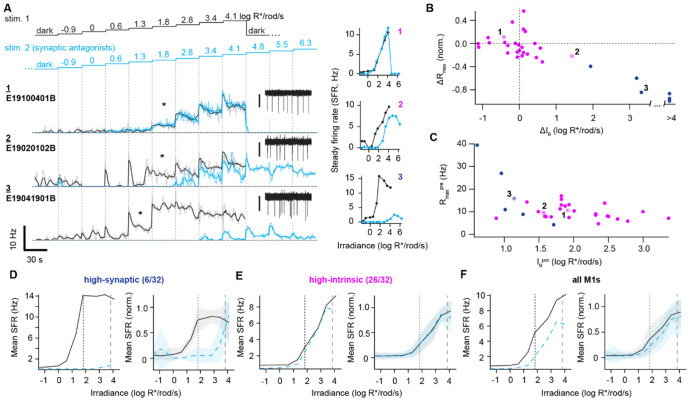
Diverse synaptic and intrinsic drives across M1 ipRGCs account for circadian photoentrainment at and above threshold **A**. *Left*, Spike rates of three example M1s (rows) during a staircase stimulus (460 nm) delivered in physiological conditions (black) and then repeated with synaptic antagonists (cyan; with higher intensities to probe melanopsin responses further). 1-s bins and a 5-s moving average are represented by light/dotted lines and dark/solid lines, respectively. Stimuli with intensities (log R*/rod/s) shown above. Insets show 3 s of raw recordings taken during one step (asterisks) of each cell’s first staircase presentation; scale bars: 40 pA (**1**) and 30 pA (**2,3**). *Right*, Intensity-firing (I-F) relations for the three example cells (firing rate during the last 10 s of each step). Loose-patch recordings, 35° C. **B**. Difference in maximum steady firing rate ($\Delta R_{\max}$, normalized to the pre-antagonist firing rate) versus the difference in intensity threshold (ΔI_θ_) between physiological and synaptic antagonist conditions. Clustering gave a “high-synaptic” group (blue; 6 of 32 cells in which antagonists had a large effect on both parameters) and a “high-intrinsic” group (magenta; 26 cells in which they did not). Sample cells from **a** are marked; cell 3 is high-synaptic cluster, whereas cells 1 and 2 are high-intrinsic. **C**. Maximum steady firing rate plotted against threshold intensity for M1s preceding the addition of synaptic antagonists ($R_{\max}$ vs. I_θ_; same cells as in **B**). Cluster memberships and example cells are designated. High-synaptic cells tend to have lower thresholds and higher maximum firing rates. **D**. *Left*, Average I-F relations before (solid black) and after (dashed cyan) addition of synaptic antagonists for high-synaptic M1s (6 cells). *Right*, Normalized I-F relations (minimum is 0 and maximum is 1 for each cell). Shaded regions are ±1 SD. The intensities at which >80% of wild-type and *Gnat1*^*−/−*^ mice photoentrained^2^ are marked by vertical dotted and dashed lines, respectively. **E-F**. As in **D**, but for high-intrinsic M1s (**E**, 26 cells) and all M1s (**F**).

To investigate how synaptic and melanopsin drives produce irradiance encoding by M1s, we made somatic loose-patch recordings (35 °C) and delivered staircases of ascending intensity (Figure 2A). The first staircase covered a relatively broad range of intensities while allowing intrinsic photosensitivity to recover from light adaptation in a practical interval (10 min; 30-s steps from −0.9 to 4.1 log R*/rod/s). We applied synaptic antagonists in this interval, then delivered a second staircase that was identical to the first but continued to higher intensities (up to 4.8-6.3 log R*/rod/s, spanning the intrinsic dynamic range of many M1s^27^). In some cells, we delivered three staircases (the third with an extended range) to test for intrinsic response stationarity (which was confirmed; Supplementary Figure 4). This protocol allowed comparison of individual M1 outputs in physiological conditions (first staircase) and with intrinsic photosensitivity isolated (second staircase).

In physiological conditions, the mean threshold intensity that modulated steady firing (I_θ_) by ≥1 Hz was 1.47 ± 0.60 log R*/rod/s (39 cells), consistent with the flash thresholds reported above for a different sample of M1s (0.50 log R*/rod; dividing by the 0.2-s rod integration time gives an estimated step threshold of 1.20 log R*/rod/s). Across the sample, step I_θ_ covered a ~575-fold range (0.15-2.91 log R*/rod/s), comparable to the aforementioned range of flash thresholds. In synaptic antagonists, I_θ_ doubled to 1.83 ± 0.94 log R*/rod/s (p = 0.016, 30 of the 32 cells that completed the second staircase). This is similar to previous estimates of intrinsic thresholds from us and others^23,27^. Intrinsic I_θ_ values spanned a ~4900-fold range, from 0.54 to 4.23 log R*/rod/s. This is a lower bound; 2 cells had undefined I_θ_ because their steady firing was not modulated (i.e., ≥1 Hz change) by the stimulus in antagonists. Thus, synaptic input lowers and regularizes the thresholds of M1s, potentially allowing the population to signal with higher fidelity when light is scarce. Turning to the upper end of the intrinsic intensity-firing (I-F) relation for the subset of cells that received the maximum intensity (6.3 log R*/rod/s), we found that spiking was silenced in most (15 of 22 cells). This is consistent with our prior work demonstrating that M1s are tuned to light intensity by depolarization block^27^.

Comparing physiological and antagonist conditions, differences in I_θ_ and the maximum steady firing rate ($R_{\max}$) were correlated across all M1s examined (Spearman r = −0.68, p = 2×10^−4^), suggesting that synaptic input influences M1s across their dynamic range. Nevertheless, ΔI_θ_ and $\Delta R_{\max}$ were small for most M1s and drastic for a minority; plotted by these parameters, cells formed a continuum with two densities (Figure 2B). To examine this variation further, we considered cells in terms of heuristic, “high-intrinsic” and “high-synaptic” groups (using k-means clustering). In physiological conditions, high-synaptic cells had lower I_θ_ (0.62 ± 0.37 vs. 1.55 ± 0.55 log R*/rod/s, p = 3.5×10^−4^), as expected from stronger rod drive, and tended to have higher $R_{\max}$ (Figure 2C). Variation in the degree of rod drive is consistent with a prior study using a single light intensity^26^. It is also evident in melanopsin-null M1s: Steady firing was practically absent in some (suggesting a normally high dependence on intrinsic photosensitivity) and tracked intensity across orders of magnitude in others (revealing a high degree of sustained synaptic input; Supplementary Figure 5). Some M1s act more like autonomous photoreceptors and others more like conventional RGCs.

## M1 ipRGCs account for the dynamic range of circadian photoentrainment

The threshold of circadian photoentrainment is estimated to be 0.37 log R*/rod/s and relies on rod signals carried by ipRGCs^2,5^. High-synaptic M1s are activated at this irradiance, raising their firing rate by 0.80 ± 0.63 Hz (6 cells). This activity depends on synaptic input, as the firing rate falls to −0.3 ± 0.15 Hz in antagonists (p = 0.002, Figure 2D). Indeed, at this irradiance, high-intrinsic M1s elevate their firing by only 0.01 ± 0.97 Hz (Figure 2E). At a higher irradiance of 1.86 log R*/rod/s, >80% of mice photoentrain^2^, likely due to input from both high-synaptic (13.7 ± 13.3 Hz above baseline, 6 cells) and high-intrinsic M1s (2.2 ± 1.6 Hz, 26 cells; Figure 2D-F). At even higher irradiances, high-synaptic M1s provide little additional signaling because of saturation (maximum rate of 13.8 ± 10.4 Hz); meanwhile, high-intrinsic M1s can fire up to 8.7 ± 3.6 Hz (reached at 4.13 log R*/rod/s). Hence, synaptic drive of the most sensitive M1s can account for behavioral threshold, and the progressive recruitment of additional M1s and intrinsic photosensitivity can explain how photoentrainment strengthens as light intensifies.

With rods inactivated, a hundred-fold higher irradiance (~3.86 log R*/rod/s) is required to photoentrain >80% of mice^2^. This elevation is likely due to loss of drive to high-synaptic but not high-intrinsic M1s; at 1.86 log R*/rod/s, synaptic antagonists diminish firing of the former to 0.19 ± 0.47 Hz (p = 0.002) but hardly change that of the latter (p = 0.30; Figure 2D-E). The light responses of high-intrinsic M1s may explain photoentrainment of rod-inactivated mice; at 3.86 log R*/rod/s, in antagonists, they fire strongly (7.7 ± 3.5 Hz) while high-synaptic cells fire weakly (0.61 ± 0.74 Hz). Therefore, M1 heterogeneity can explain long-standing behavioral data on photoentrainment thresholds of different genotypes^2^, with the critical synaptic and intrinsic drives being differentially distributed across the population.

## Temporal integration by M1s resembles that of circadian phase shifting

**Figure 3.**
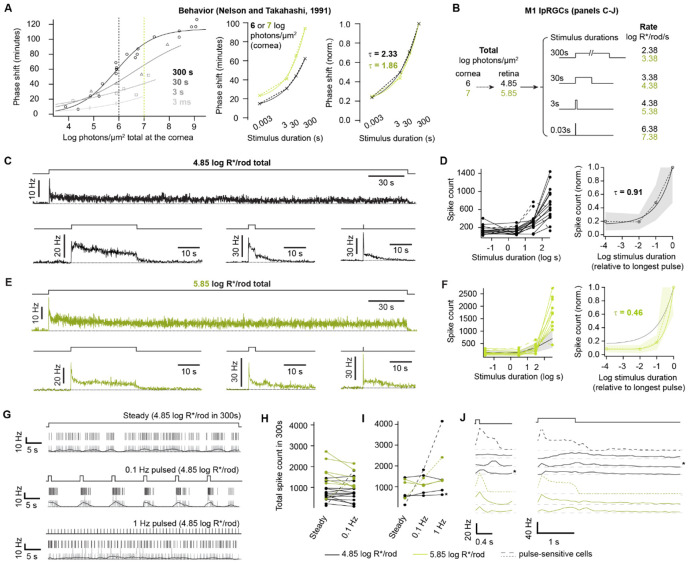
Precise temporal integration by M1 ipRGCs **A**. *Left,* Behavioral phase shifts defined by Nelson and Takahashi^3^ to various photon counts (per unit area) delivered over 300 s (circles), 30 s (triangles), 3 s (squares), or 3 ms (diamonds). Fits are modified Naka-Rushton functions^3^. *Center,* Duration-response (D-R) relations for phase shifting at two photon counts at the cornea: 6 (black) and 7 (green) log photons/μm^2^ (Methods). Each D-R relation is fit with a single exponential. *Right,* Normalized D-R relations with the time constants of their single exponential fits. For a given photon count, the phase shift varies only ~5-fold for a 10^5^-fold change in stimulus duration, indicating precise temporal integration. **B**. The stimulus suite, modeled on those of ‘a,’ used to examine the precision of temporal integration by M1s. Pulses deliver the same photon count (per unit area) but over different durations. Briefer pulses have higher photon flux densities. Two photon counts are tested, which yield 4.85 (black) and 5.85 (green) log R*/rod at the retina (broadband light). These photon counts correspond to 6 and 7 log photons/μm^2^ at the cornea (Methods). **C**. Average spike firing rates of M1s in response to 4.85 log R*/rod delivered over 300 s (17 cells), 30 s (15), 3 s (15), and 30 ms (16). Stimulus monitor at top. The stimulus order was randomized for each cell. The inter-stimulus interval in darkness was >300 s. Loose-patch recording, 35 °C, no synaptic antagonists. **D**. *Left,* Duration-firing (D-F) relations for all M1s in **c** (individual responses are shown in Supplementary Figure 6). Spikes were counted from the onset of the stimulus to the return of spike rate to baseline and corrected for firing rates in darkness. Most relations were fit by a single exponential (τ = 0.86 ± 0.27 log units, 11 of 17 cells). Dashed traces indicate unusual, non-monotonic D-F relations. *Right,* D-F relations were normalized to maximum and averaged (solid line; shading is ±1 SD). A single exponential is fit with τ = 0.91. **E**. As in **C**, for a different set of M1s (10 cells) given 5.85 log R*/rod over different durations. **F**. As in **D** but for 5.85 log R*/rod stimulus suite. The single exponential fit to the average has τ = 0.46. For comparison, the average D-F (left) and single exponential fit to the average (right) are shown for the 4.85 log R*/rod stimulus suite. **G**. Spike firing of an example M1 during stimuli with different temporal structures but delivering the same R* count (4.85 log R*/rod in 300 s). *Top,* A steady, 300-s step (2.38 log R*/rod/s). *Center,* 0.1-Hz pulses (1-s duration, 3.38 log R*/rod/s each). *Bottom,* 1-Hz pulses (0.1-s width, 3.38 log R*/rod/s each). Response excerpts are taken ~90-150 s after stimulus onset, and each shows the stimulus (top), spike rasters (center), and spike rate histograms (0.1-s bins in grey and 0.5-s moving average in black). Loose-patch recording, 35 °C, no synaptic antagonists. **H**. Total M1 spike counts for the 300-s uniform step and 0.1-Hz pulsed stimulus, for 4.85 (black) or 5.85 (green) log R*/rod. Spike counts differed between R* counts (p = 0.007 and p = 0.006 for 4.85 and 5.85 log R*/rod, respectively) but not between temporal structures (p = 0.70 and 0.53 for steady and 0.1 Hz, respectively). **I**. A subset of 7 M1s received all three stimuli depicted in **G**, with total R* counts of 4.85 log R*/rod (black) or 5.85 log R*/rod (green). Two cells (dashed lines) preferred higher temporal frequencies, while most (solid lines) were insensitive to temporal structure. The example cell in **G** is marked with an asterisk. **J**. Pulse-triggered average spike rates of the 7 cells in **i**, given the 1- and 0.1-Hz stimulus (left and right, respectively; 0.1-s bins). For the 1-Hz stimulus, only the first 60 pulses are averaged to minimize any effect from adaptation. Shaded areas show the pulse timing. The example cell in **G** is marked with an asterisk.

Phase shifting of circadian behavior follows the total number of photons received and is highly insensitive to the timing of their reception^3^. We adopted the stimulus paradigm that Nelson and Takahashi used to make this discovery, illustrated in Figure 3. We delivered a given photon count over different durations (30 ms, 3 s, 30 s, and 300 s). We began with a stimulus producing 4.85 log R*/rod at the retina (close to the 6 log photons/μm^2^, measured at the cornea, used behaviorally; Figure 3A-B). We chose this count for two main reasons. First, dividing it by the tested durations gives a range of intensities in which synaptic and intrinsic drives are likely to contribute. The shortest duration is roughly equivalent to moderate daylight (6.4 log R*/rod/s at the cornea) and the longest to twilight (2.4 log R*/rod/s). Second, these intensities are subsaturating for circadian phase shifts. We randomly interleaved the different durations, allowed 5-10 minutes of dark adaptation between each trial, and recorded the spiking of M1s using loose-patch electrophysiology (35 °C). Prior to delivering these stimuli, we examined threshold flash responses to estimate the degree of synaptic input received by each cell (Supplementary Figure 2F).

Each pulse duration evoked different patterns of spiking that varied across cells and for the sample mean (Figure 3C, Supplementary Figure 6). We examined the relation of stimulus duration (in units of log s) and the M1 response (total spike count, measured from the onset of the stimulus to the return to baseline). This duration-firing (D-F) relation would be flat for perfect temporal integration. We found that the D-F relations of individual cells, from 30 ms to 300 s, were usually well-described by a single exponential (11 of 17 cells, Figure 3D; see below regarding exceptions). The mean time constant (τ) was 0.86 ± 0.27 log s (11 cells). The normalized sum of all D-F relations is fit with a τ of 0.91 log s. Across the 10^4^-fold span of tested durations, the population spike count varied by only ~5-fold. The spike count changed most steeply from 30 to 300 s; however, in this 10-fold range, the change was only ~2.7-fold. This degree of temporal integration approaches but does not quite reach that observed for behavioral phase shifting (behavioral τ is shallower at 2.33 log s, Figure 3A; see below).

To examine temporal integration of more intense stimuli, we tested a photon count that produced 5.85 log R*/rod (Figure 3E). This higher photon count produced higher spike counts (Figure 3F), reflecting intensity coding by M1s. The D-F relation was also steeper (Figure 3F; population τ = 0.46 log s). Nevertheless, across the 10^4^-fold span of durations, the population spike count varied by only ~12-fold; in the steep region from 30 to 300 s, this variation was ~5.5-fold. Temporal integration is lower at this higher photon count but remains effective even though synaptic and intrinsic mechanisms are driven at different points in their dynamic ranges.

Nelson and Takahashi also observed precise temporal integration of stimuli that differed in their temporal structure; for example, a given photon count received steadily or in trains of discrete pulses^4^. We compared M1 spiking for constant and pulsed stimuli that produced 4.85 or 5.85 log R*/rod in 300 s (Figure 3G-J). We chose this duration because it is especially effective at driving behavioral phase shifts^3,4^. We gave pulses at 0.1 Hz (14 and 7 cells at the low and high photon counts, respectively). We also tested 1 Hz in a subset (4 and 3 cells at these photon counts). At both photon counts, all cells followed the 0.1-Hz stimulus to a degree, and all but one followed the 1-Hz stimulus. Different cells spiked with different patterns, indicating variation in the relative contributions of synaptic and intrinsic drives. Despite these temporal dynamics, at each photon count, most cells showed little difference in total spike count among steady and pulsed stimuli (Figure 3H-I). This was the case whether synaptic drive appeared low (the cell that failed to follow 1-Hz pulses) or high (cells showing large, fast transients at pulse onsets), indicating that diverse synaptic and intrinsic balances support precise temporal integration. The higher photon count also produced a higher population spike count across both steady and 0.1-Hz pulsed stimuli, consistent with M1s coding the overall light intensity.

Two exceptions in our sample strongly preferred the pulsed stimuli, firing more spikes with higher stimulus frequency (steady, 0.1, and 1 Hz; Figure 3H-I, **dashed traces**). These cells had low flash thresholds (15-34^th^ percentile), consistent with a high-synaptic classification. While anecdotal, this observation suggests that some M1s may account for one aspect of circadian photoregulation but not another—in this case, threshold but not temporal integration.

## Intrinsic and synaptic origins of temporal integration by M1 ipRGCs

**Figure 4.**
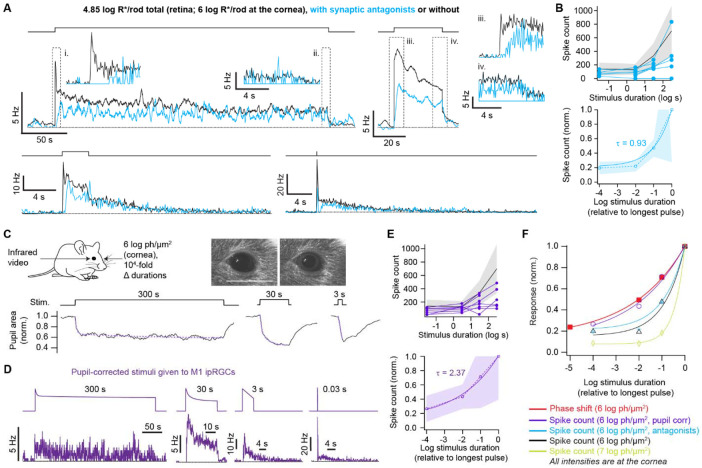
Synaptic input and pupillary constriction tune the temporal integration of M1 ipRGCs **A**. Average spike firing rates of M1s in synaptic antagonists (cyan) and given 4.85 log R*/rod over 300, 30, 3, and 0.03 s (5-8 cells per stimulus duration). Stimulus monitors at top. Responses without antagonists are shown for comparison (black; same data as in Figure 3C). Insets show the beginning (left) and end (right) of these responses on an expanded time base. Firing rates are shown as 0.5-s moving averages for clarity, and insets are binned at 0.1 s to resolve transients. Loose-patch recording, 35 °C. **B**. *Top,* Individual D-F relations of the M1s in synaptic antagonists (cyan, 9 cells). The average D-F for M1s without antagonists is provided for comparison (mean and S.D. in black and gray, respectively). *Bottom,* D-F relations of M1s in synaptic antagonists were normalized to their maxima and then averaged (dashed line). Single exponential fit, τ = 0.93 (solid line). Shading is ±1 SD. **C**. *Top,* Light pulses were delivered to one eye (4.85 log R*/rod at the retina) and pupil constriction was measured in the contralateral eye (Methods). Individual video frames show the pupil of an example mouse in darkness (left) and at the peak of its response to a 300-s light pulse (right). Scale bar: 5 mm. *Bottom,* Pupil diameter, normalized to its dark-adapted level, for three stimulus durations (black; average of 8 trials for 4 mice). A double exponential fit is overlaid for 300 s, and single exponential fits for 30 s and 3 s (violet). **D**. Light pulses were attenuated according to pupil constriction to make pupil-corrected stimuli (i.e., the original stimulus was multiplied by the normalized pupil area over time), which were given to M1s. Average spike rates are given for each pupil-corrected stimulus (300, 30, and 3 s) and an uncorrected 30-ms stimulus (a comparable stimulus given to the animal was complete before pupil constriction began). Each trace is an average of 6-7 cells. **E**. *Top,* D-F relations for single M1s in **I** (violet, 7 cells). The average D-F from the pupil-uncorrected, 4.85 log R*/rod group is shown for comparison (black and grey; same data as in Figure 3D). *Bottom,* D-F relations of the pupil-corrected group were normalized to their maxima and averaged (dashed line). Single exponential fit (solid line), τ = 2.37. Shading is ±1 SD. **F**. Population D-F relations of all four experimental M1 groups. A behavioral phase-shifting D-R from Nelson and Takahashi is plotted for comparison (6 log photon/μm^2^, equivalent to the 4.85 log R*/rod given to M1s; see Figure 3B). With pupil correction, temporal integration by M1s resembles that of animal behavior. Light intensities are given in terms of corneal intensities in units of photons/μm^2^ to make comparisons between these data sets more straightforward (Methods).

To investigate the synaptic and intrinsic origins of precise temporal integration, we repeated the experiments of Figure 3 (delivering light to produce 4.85 log R*/rod, in the form of continuous pulses of different durations) in synaptic antagonists. The mean transient response at light onset was smaller and slower than in physiological conditions; spike latencies <1 s were practically absent for the 30- and 300-s stimuli (Figure 4A-B). Cells were also insensitive, with one responding only to the most intense (30-ms) stimulus. By contrast, all cells responded to all durations in physiological conditions (see above). Spike counts were also lower across all stimulus durations. These observations are consistent with melanopsin phototransduction’s kinetics, sensitivity, and complementation by synaptic inputs.

Despite these effects of synaptic antagonism, the shape of the population D-F curve was essentially unchanged (Figure 3D, 4B, and 4F)—the population τ was 0.93 with antagonists and 0.91 without. In both conditions, across the 10^4^-fold range of durations, the population spike count varied by ~5-fold; between 30 and 300 s, it varied by ~2-fold. Hence, synaptic inputs largely result in a scaling of the population spike count set by intrinsic activity, despite the different sensitivities and kinetics of these two drives, to support precise temporal integration.

One exception to this scaling is that, at the end of the 300-s step, the population spike rates without and with synaptic antagonists became largely indistinguishable (Figure 4A). Over long time scales, temporal integration relies largely on melanopsin phototransduction.

A handful of cells fired strongly at stimulus onset, maintained unusually high firing rates during the 30-s stimulus, and were scarcely active toward the end of the 300-s stimulus (3 of 27 cells in physiological conditions, high and low photon counts represented). Among these apparent high-synaptic cells were the two that preferred pulsed stimuli (see above and Figure 3). Such cells tend to flatten the population D-F relation, which swings upward at longer durations. Omitting them from the sample yields a slightly steeper relation (τ = 0.77 log s for the low intensity illustrated in Figure 3C-D). These rare M1 responses underscore the idea that different balances of synaptic and intrinsic drives across the population support precise temporal integration.

## Pupil constriction raises temporal integration of M1 ipRGCs to the behavioral level

We hypothesized that temporal integration is more precise *in vivo* because longer stimuli are attenuated by pupillary constriction, reducing M1 spike counts and flattening the D-F relation. To test this idea, we measured the pupil of behaving, wild-type mice while giving light producing 4.85 log R*/rod over different durations (Figure 4C). Pupillary constriction was undetectable during the briefest stimulus. For the other three durations, we measured the time courses of constriction and designed stimuli that mimic their attenuations of light intensity (Figure 4D). We delivered these pupil-corrected stimuli to M1s in the *ex vivo* retina under physiological conditions. Temporal integration was more effective for these stimuli (population D-F relation τ of 2.37 log s, 7 cells; Figure 4E-F). Across the 10^4^-fold difference in stimulus durations, the population spike count varied by ~3.8-fold; between 30 to 300 s, it varied by 1.4-fold. The D-F relation closely matched that of behavioral phase shifting measured by Nelson and Takahashi (Figure 4F). Pupil constriction appears to increase the precision of circadian photoregulation by tuning the temporal integration of M1s.

## DISCUSSION

M1 ipRGCs are essential for circadian regulation: they provide most retinal innervation of the brain’s principal clock, are obligate relays of rod and cone signals to this structure, and set the clock even with only their intrinsic photosensitivity^14,15^. We have investigated how the functional properties of M1s support this role.

To this end, we developed a bioluminescence method to identify these rare neurons without desensitizing the retina. This method facilitates investigation of cellular and circuit mechanisms in a behaviorally relevant context. We introduce a mouse line and viral vectors for reporter expression, the substrate is readily available, and suitable cameras are more economical than 2-photon fluorescence systems. Reporter expression level, substrate concentration, and detection sensitivity may be adjusted according to the needs of specific studies. Incorporating reporters of different spectra and higher emission^18,38^ could increase the accessibility and flexibility of the approach.

M1s are understood to encode light intensity^10,39,40^. The apparent simplicity of the task contrasts with the complexity of M1 light responses at the level of single cells (e.g., adaptation, depolarization block, and heterogeneity) and of their microcircuitry^24,26,27,33,41-44^. We find that these responses account for two hallmarks of clock photoregulation^3,4^ observed in animal behavior. One is its dynamic range, with an elevated threshold permitting vision in dim light without circadian resetting. The most sensitive M1s account for this threshold, and other M1s are recruited as light intensifies. Another is the clock’s precise temporal integration, which sums light over minutes to promote the encoding of ambient illumination, a proxy for solar elevation. Response complexity supports this process across orders-of-magnitude changes in environmental irradiance. For example, higher light intensities produce more adaptation and depolarization block to trim spike outputs, and different intensities engage these mechanisms at distinct levels in varied subsets of M1s (Supplementary Figure 7).

We find that pupil constriction tunes the M1 population such that its precision of temporal integration matches that observed behaviorally (Figure 4F). Pupil constriction is stronger even than synaptic input in shaping the temporal integration of M1 outputs (Figure 4F). The level of temporal integration in behavioral phase shifting is constant across a broad range of irradiances^3^ (Figure 3A). This suggests that pupillary constriction operates accurately over this range to support circadian photoregulation, as it does for visual perception^45^. Pupil constriction, like circadian photoregulation, is largely driven by M1s^10^—the distinct processes influenced by these cells appear to be highly coordinated.

M1s are classified as a single cell type by physiological, morphological, and molecular parameters. Variations in these parameters support subdivisions of M1s, though these groups do not cohere across parameters^14,24,26,27,44,46-48^. In our sample of M1s, some act more as photoreceptors and others as relays for rod/cone signals. For those with identified locations, the former were enriched in the ventral retina (10/10 identified ventral cells were high-intrinsic, versus 3/5 in the dorsal retina) and the latter in the dorsal retina (2/5 identified dorsal cells were high-synaptic, vs. 0/10 in the ventral retina), consistent with dorsal M1s showing lower melanopsin immunoreactivity and higher dendritic complexity^26,44,47^. Future studies may provide a logic to these variations. They may also determine if our high-synaptic and high-intrinsic grouping is more than an operational definition, which we made to facilitate investigation of how cellular heterogeneity supports behavior. Such studies are likely to require high-throughput methods because M1s are rare and varied, and we have observed that even outliers in the population may make important contributions.

Our connections of cellular mechanisms to behavior have caveats. First, the threshold of circadian photoentrainment was tested with longer light exposures than those used here^2^; while the *ex vivo* techniques we employ are necessary for the mechanisms in question, a single experiment has limited duration. The comparison is likely fair because the firing of an ipRGC is stable within its dynamic range^27,33,42,49^. Second, temporal integration of circadian phase shifting was defined using the hamster^3,4^, while our experiments required expanding upon tools that have been established over decades for mice. While species comparisons require care, we found exquisite agreement between the two data sets. This concern is also mitigated by the conservation of M1 signaling mechanisms between mice and an even more distant species, the macaque monkey^50^. Aligning knowledge across timescales and species continues to be a major undertaking in the field.

Sensory neurons encode salient aspects of the environment. This work indicates that M1s produce highly customized information for clock regulation. Performing these computations at this early stage makes them available to the dozens of brain regions that these neurons innervate^51^, potentially endowing them with features that have been considered signatures of the circadian system.

## METHODS

### Generation of mice for Cre-dependent expression of nano-lantern from the ROSA26 locus (*ROSA26*^*FLEX-NL*^)

Mice were generated using a protocol for insertion of NL into the ROSA26 gene locus using CRISPR/Cas9^53^ (Supplementary Figure 1). The guide RNA was 5’-ACTCCAGTCTTTCTAGAAGA - 3’ (Millipore Sigma).To validate the line, a NL^+/+^ adult mouse was anesthetized and euthanized. ~0.5 g of liver tissue was removed and digested overnight at 55° C in 4 ml of 20 mM Tris (pH 8), 5 mM EDTA (pH 8), 400 mM NaCl, 1% SDS, and 400 μg/ml Proteinase K (Qiagen 19131). Digestion was quenched at 80° C for 10 minutes. The sample was washed in buffer-saturated phenol (Thermo Fisher Scientific, 15513-39), phenol-chloroform-isoamyl alcohol (25:24:1, Thermo Fisher Scientific, 15593031), and twice in chloroform-isoamyl alcohol (24:1, Millipore Sigma, 25666; all volumes were 4 ml). For each wash, the sample was inverted (10×), incubated (10 min, ~23° C), and centrifuged (17×g, 1 min; room temperature, ~23° C); the aqueous phase was isolated for the next wash. DNA was precipitated by mixing the final aqueous phase with 4 ml of −20° C ethanol and incubated for 40 min (−20° C ) . DNA was transferred via a glass hook into 1.5 ml of 70% ethanol in double-distilled water (4° C) and incubated on ice (15 min). The ethanol solution was decanted and the DNA was dried at 55° C (~20 minutes). It was then suspended in Buffer EB (10 mM Tris-Cl, pH 8.5, 500 μl; Qiagen 19086) via rocking overnight (4° C). ~23 μg of DNA was obtained (OD_260/280_ of 2.02 and OD_260/230_ of 2.16, measured using a NanoDrop Spectrophotometer; Thermo Fisher Scientific).

Primers flanking the *ROSA26* locus were designed using Primer Blast (NCBI) and manual optimization: Forward, 5’-GTCCTGACCCAGGGAAGACATTAAAAA-3’, and Reverse, 5’-AACCCAAAGAAGTGCTTCTGAGTATAAA-3’. Primers were acquired at 100 μM concentration in 10 mM Tris, pH 8.5 (Azenta). Four 50-μl PCR reactions, each including ~200 ng of template DNA, were performed using PrimeSTAR GXL DNA polymerase (Takara, R050A), following manufacturer guidelines. PCR products were pooled, cleaned, and concentrated (Zymo, D4003) in 15 μl of Buffer EB. A ~142 ng/μl product was obtained (OD_260/280_ of 1.88 and OD_260/230_ of 2.21). 1 μl of this product was diluted in 14 μl of nuclease-free water and mixed with 3 μl of 6X Gel Loading Dye (New England Biolabs, B7025S). The mixture was run on 0.7% agarose gel with 1X SYBR Safe (Thermo Fisher Scientific, S33102) alongside a DNA ladder (New England Biolabs, N3200S), then imaged (Bio-Rad GelDoc XR). A single band was observed at ~10 kb, matching the expected size of the PCR product (9,992 bp, Supplementary Figure 1).

10 μl of the concentrated DNA product was submitted for long-read, end-to-end sequencing (Oxford Nanopore, Plasmidsaurus). Over 400,000 base pair reads, from fragments of 9-10 kb length, were used to generate a consensus sequence. This sequence matched that of the construct almost perfectly. The only deviations within the knock-in sequence were outside of open reading frames or regulatory elements. The remaining misalignments are likely variations in the mouse genetic background (Supplementary Figure 1).

### Mouse lines

Most experiments used a transgenic mouse line expressing Cre from the melanopsin (*Opn4)* locus of a BAC transgene (*Opn4*^*BAC-Cre*^; endogenous *Opn4* alleles were not targeted)^54^. Transduction of these mice with Cre-dependent viral vectors gave robust labeling of ipRGCs. However, crossing these mice with *ROSA26*^*FLEX-NL*^ did not. Experiments on M4 ipRGCs used a line in which the gene encoding Cre recombinase replaced one allele of *Opn4*^19^. It was combined with *ROSA26*^*FLEX-NL*^ (*Opn4*^*Cre/+*^; *ROSA26*^*FLEX-NL/+*^). Loss of one *Opn4* allele is inconsequential in these experiments because they concerned rod-driven thresholds, which this study found to resemble those of wild-type M4 ipRGCs^21,22^. Replacing both alleles of melanopsin (*Opn4*^*Cre/Cre*^) produced melanopsin-null mice. Experiments with rod or cone inactivation used *Gnat1*^*−/−*^ or *Gnat2*^*−/−*^ mice, respectively^36,37^. These knockouts disable phototransduction, locking rods and cones in the dark state without degeneration. All mouse lines were maintained on a C57BL/6J background. Mice of both sexes were used. Ages were P30-P180.

### Generation of nano-lantern viruses

To generate an AAV vector for Cre-dependent nano-lantern (NL) expression (AAV-FLEX-NL), the NL cDNA was PCR amplified from a pcDNA vector. It was subcloned into an AAV vector, inverted, between alternating loxP and lox2272 sites to generate a FLEX cassette. The construct was flanked by ITR sequences and contained an EF1α promoter, WPRE sequence, and HGH polyadenylation signal. To generate a Cre-independent AAV vector for NL expression (AAV-NL), loxP and lox2272 sites were removed and inversion of the NL cassette reversed (Genscript). Both vectors were validated with sequencing. Viruses (AAV2/1) were produced by the viral core of the Intellectual Disease and Developmental Disorder Center of Boston Children’s Hospital.

### Stereotaxic injection of viruses

Mice (>P60) were anesthetized with isoflurane, fixed in a stereotaxic frame, and prepared for sterile surgery. A midline incision revealed the skull and a craniotomy was made. *Opn4*^*BAC-Cre*^ mice were injected with a 1:1 mixture of AAV2/1-FLEX-NL (4.4×10^13^ gc/ml) and AAV2/1-CAG-H2B-tdTomato (Addgene #116870, 2.4×10^13^ gc/ml); nuclear-localized tdTomato was used to check the injection site. In experiments using *Gnat1*^*−/−*^ or *Gnat2*^−/−^ mice (which did not express Cre), AAV2/1-NL was used instead (2.9×10^13^ gc/ml). Viral injections were targeted to the SCN with the following stereotaxic coordinates, all measured from bregma: 0.88 mm lateral, 0.46 posterior, 5.90 mm travel distance. These injections were performed at a slight angle (6° degrees from vertical, pointing toward the midline) to avoid tracking into the third ventricle. Injection pipettes were pulled from filamented borosilicate glass (1.0-mm outer diameter) and had a ~60-μm tip diameter. Once the pipette was lowered to its target, it was kept in place for 5-10 minutes before infusing a total volume of 500 nl at a rate of 0.1 ul/min. After infusion, 5-10 min was given before slowly withdrawing the pipette from the brain. The wound was closed and mice recovered from surgery and meloxicam or Ethiqa XR was given for analgesia. Viral expression was first visible ~2 weeks following injection.

### Electrophysiology solutions

The extracellular solution was bicarbonate-buffered Ames' medium (Sigma or US Biological) or “ionic” Ames’ medium^24,33^ (in mM: 120 NaCl, 22.6 NaHCO_3_, 3.1 KCl, 0.5 KH_2_PO_4_, 1.2 CaCl_2_, 1.2 MgSO_4_, and 6 glucose), both equilibrated continuously with carbogen (95% O_2_/5% CO_2_) for a pH of 7.4. Synaptic transmission was blocked with an established cocktail^24,25,27,33,50^: 3 mM kynurenic acid, 100 μM picrotoxin, 10 μM strychnine, and 100 μM D, L-AP4. The internal solution for perforated-patch recording was (in mM) 110 K-methanesulfonate, 13 NaCl, 2 MgCl_2_, 10 EGTA, 1 CaCl_2_, 10 HEPES, 0.1 Lucifer Yellow, and 0.125 amphotericin B; pH 7.2 with KOH. Amphotericin B was dissolved in DMSO to make 100× aliquots, and stored in the dark at −20° C for up to two weeks. A liquid junction potential of +7 mV has been corrected. The pipette solution for loose-patch recording was HEPES-buffered Ames' medium (in mM, 140 NaCl, 3.1 KCl, 0.5 KH_2_PO_4_, 1.2 CaCl_2_,1.2 MgSO_4_, 6 glucose, 10 HEPES; pH 7.4 with NaOH). Coelenterazine-h stock was made at 200× in 100% ethanol under infrared light, and stored in darkness at −80 C for up to six months.

### Retina preparation

Mice were dark adapted for >1 hour prior to experimentation, and subsequent procedures took place under infrared illumination using infrared-visible converters that were head- and microscope-mounted. Dim, indirect red light was used sparingly as needed. Mice were deeply anesthetized with Avertin, enucleated, and euthanized. The retina was isolated at room temperature in carbogenated Ames’ medium (see above) and vitreous humor removed. The retina was flattened with peripheral cuts and mounted on a glass coverslip coated with poly-L-lysine, RGC side up. Occasionally, to increase the number of recordings per animal while minimizing total light exposure during experimentation, the retina was cut in half, each piece was mounted on a separate coverslip, and one was incubated in carbogenated Ames’ medium at room temperature until use.

### Cell imaging and identification

Before electrophysiological recording, the mounted retina was transferred to a perfusion chamber containing carbogenated Ames’ medium. The medium was replaced with 1 ml of carbogenated Ames’ medium containing 20-50 μM coelenterazine-h. The chamber was immediately moved to the electrophysiology rig and superfusion with carbogenated Ames’ medium was initiated. Detectable NL bioluminescence decayed over tens of minutes. During this period, as many NL-positive cells as possible were identified using an EM-CCD camera (Hamamatsu ImageEM) and the inner limiting membrane over their somata removed mechanically. Recordings were only initiated once the bioluminescence from these cells was no longer visible. After the conclusion of recording from a given retina, NL fluorescence was used to observe cell morphology and dendritic stratification. M1 cell identity was confirmed by the presence of dendritic stratification exclusively in the OFF sublamina (~40 microns below the ganglion cell layer). M4 ipRGCs were identified by their bioluminescence (in the *Opn4*^*Cre/+*^; *ROSA26*^*FLEX-NL/+−*^ mouse line) and large somata^55^. Control M4 and other α-RGCs were targeted for recording by their soma size under infrared illumination; at the conclusion of the flash threshold determination, a 500-ms pulse of light was delivered to determine the α-RGC subtype (ON or OFF, sustained or transient^56^).

### Electrophysiology

For loose-patch recording, pipettes (2-5 MΩ) were wrapped with parafilm and seal resistances were 10-30 MΩ. Recordings were conducted in voltage clamp with a holding potential of 0 mV. Recordings were discarded if the spontaneous spike rate or spike amplitude became unstable. Cells were also excluded from synaptic threshold measurements if their threshold firing showed evidence of melanopsin involvement (i.e. if firing continued to increase for 1-2 seconds following the flash, making fast synaptic responses impossible to separate; 4/36 cells). All loose-patch recordings were performed at 35° C. For perforated-patch recording, pipettes (3.5-6 MΩ) were wrapped with parafilm to reduce capacitance. Seal resistances were >10GΩ. Series resistances (typically <60 MΩ) were monitored but not compensated. Perforated-patch recordings were discarded if the initial seal resistance was <10 GΩ, access resistance was >100 MΩ, holding current at −80 mV was unstable, or any of these parameters changed abruptly. All perforated-patch recordings were performed at 23° C for additional stability.

### Pupillometry

Animals were implanted with a headpost^57^, recovered for >1 week, and were acclimated to the experimental setup. For experiments, they were dark adapted for >2 hrs and all subsequent procedures were done using dim red headlamps that were not directed toward the mouse. Tropicamide drops were applied to the stimulated eye to dilate its pupil, but not to the contralateral eye. Light was delivered by a Ganzfeld sphere (custom, internally coated with BaSO_4_), whose output is adirectional and thus allows precise quantification of retinal illumination^25^. The sphere was placed over the stimulated eye. An infrared light and video camera were focused on the contralateral eye to measure the consensual pupil response.

Optical stimuli matched those used *ex vivo* to measure duration-firing relations for 4.85 log R*/rod (at the retina) delivered in single pulses. Instead of 30 ms, 24 ms was used due to technical constraints, but both are instantaneous (impulse stimuli) for the retinal photoreceptors. The light intensity was scaled 7.14-fold to accommodate attenuation by the eye’s optics^25,58^. Stimuli were delivered in order of increasing intensity (and descending duration). Each stimulus duration (300 s, 30 s, 3s , <30 ms) was delivered twice to each animal. The interstimulus interval was set to allow complete pupil dilation, assessed by the experimenter and then verified in analysis.

Recordings were analyzed in ImageJ using custom macros^25^. Recordings were adjusted for contrast and then binarized. The pupil was delineated by an ellipsoidal region of interest (ROI), whose maximum diameter was measured in each frame. Frames were inspected and those lacking accurate measurement were removed. Diameters were normalized to the mean in the 10-s before stimulus onset. Pupil diameters were averaged across trials and animals for each stimulus, binned (1 s), and used to calculate pupil area.

### Optical stimulation

Light sources were focused on the end of a liquid light guide for delivery to the microscope or the Ganzfeld sphere. For *ex vivo* experiments, light was collimated, sent through the microscope’s light path, focused by an objective or condenser, and centered on the soma of the recorded cell. For *in vivo* experiments, the light guide was positioned such that it was outside the animal’s field of view, such that all light entering the pupil had been internally reflected within the sphere.

All light sources were calibrated regularly using a radiometer. For broadband illumination, the spectrum’s shape was measured using a spectrometer and the intensity was scaled according to measurements with a radiometer. This procedure was used because the spectrometer was fiber-coupled and thus sensitive to the geometry of the stimulus, while the radiometer used a large, flat sensor that was insensitive. The scaling was performed by measuring the 460-nm band (10-nm width at half-maximum) with the radiometer and then scaling the spectrum accordingly, taking into consideration the attenuation within this band by the bandpass filter. Pulsed stimuli from LEDs were also verified with a fast photodiode.

#### Dim flashes.

For eliciting threshold synaptic and melanopsin responses, light from a 75-W xenon arc lamp was filtered to select wavelength and intensity, excluding infrared and ultraviolet light. Stimulus timing and intensity (20-30 ms) was controlled by an electromechanical shutter. The beam was delivered through a 40× or a 4× objective to yield a spot diameter of 300 or 2800 μm, respectively.

#### Intensity-firing and duration-firing relations.

For measuring intensity-firing relations, light from a 460-nm LED (Luminus PT120 or PT121) was bandpass filtered (460-nm center wavelength, 10-nm width at half-maximum). For duration-firing relations, a white LED was used (Luminus CBT140W). Both stimuli were collimated and delivered evenly over the whole retina using a substage condenser (as trans-illumination). Intensity was controlled with motorized neutral density filters (switching time <50 ms) as well as analog current and pulse-width modulation, using an Arduino Uno board and the ArduinoIO package for Matlab.

### Optical stimulus quantification

Stimulus intensities are often reported in units of photoactivated rhodopsin molecules per rod (R*/rod) for flashes, and R*/rod/s otherwise. The conversion from photons to R* used an effective collecting area^22^ of 0.5 μm^2^ and a standard spectral template^59^ and rhodopsin’s $\lambda_{\max}$^30^ (502 nm). For a broadband stimulus, its spectrum was multiplied by the rhodopsin template, the product integrated, and the integral multiplied by the effective collecting area. For a narrowband stimulus (10 nm width), all photons were assumed to be of the center wavelength. The intensity was multiplied by rhodopsin’s relative sensitivity at this wavelength, then multiplied by the effective collecting area. If another photopigment was considered, its template was used in place of rhodopsin’s. The $\lambda_{\max}$ values used were 358, 508, and 471 nm for short-wavelength sensitive cone photopigment, medium-wavelength sensitive cone photopigment, and the ground (R) state of melanopsin ^30,31,33,34^. For comparison with the behavioral experiments of Nelson and Takahashi^3^, their stimulus wavelength (503 nm) was referenced to the spectral template of rhodopsin (502 nm) and multiplied by the 0.5-μm^2^ effective collecting area.

Flashes and steps of light were compared using the integration time (t_i_) of the phototransduction cascade in question: 200 ms and 7.6 s for rhodopsin and melanopsin near body temperature, respectively, and 21.7 s for melanopsin at room temperature^25,33,60^. Flash intensities (photons/μm^2^) were divided by t_i_ to yield equivalent step intensities, and step intensities were multiplied by t_i_ to yield equivalent flash intensities^61^.

### Optical stimulus protocols

#### Synaptic flash thresholds.

A 4× objective was used to ensure coverage of the entire dendritic arbor of M1s and the larger M4s/ON-sustained α-RGCs^55,62^. 410 nm was used because it activates all photopigments in the mouse retina similarly^30,31,33^. Intensities were presented in ascending order (4 repetitions at each intensity, with more given if a response was not immediately apparent). Trials were analyzed offline to find the minimum flash intensity that produced a detectable response for each cell within a 500-ms window.

#### Threshold spectral sensitivity.

M1s were probed near threshold with flashes of varying intensity at 440 and 560 nm. These flashes were delivered through a 40× objective to allow corrections of cell and pipette positions, promoting recording stability, and because the spot suffices to cover the smaller M1 dendritic field^62^. Each trial consisted of 10-40 sweeps at the same flash intensity and wavelength, and wavelengths were alternated between trials. Flashes were calibrated to elicit the smallest reliable responses, usually with peaks of 2-5 pA. The average response amplitude for each trial was calculated, and the 440- and 560-nm flashes that produced the most similar responses were selected (they were within 15 ± 19%, which is 0.87 ± 1.2 pA; 20 cells). Typically, 1 or 2 matched response pairs were obtained for each cell. For each wavelength, dividing the response peak amplitude by the flash intensity gave sensitivity.

#### Measuring synaptic and intrinsic responses at threshold and above.

The initial phase of these experiments was identical to those for threshold spectral sensitivity (see above), but 500-nm flashes were used. The lowest flash intensity that produced a clear response was identified, and responses obtained for averaging. The flash intensity was then increased by 1000× and the sweep length increased to 20 s to accommodate the slower responses that were often elicited. After measuring these responses, flashes of increasing intensity (500 or 480 nm) were given until a 2-15 pA melanopsin response was visible. Occasionally, the synaptic responses near and above 1000× threshold were very small and resolved soon enough (within ~2 s) that the peak of a near-threshold melanopsin response was distinguishable; in these cases, melanopsin’s dim-flash sensitivity was measured directly from this peak. Otherwise, synaptic antagonists were applied before probing flash intensities above 1000× threshold.

#### Intensity-firing relations.

The first intensity staircase in the series consisted of 8 ascending steps, from 0.9 to 4.1 log R*/rod in ~5-fold increments, of 30-s duration. The “steady” firing rate was measured in the last 10 s of each step. While longer steps (e.g. 120 s) allow M1s to reach a more consistent steady state^27^, 30-s steps allowed 2-3 staircases to be presented during a stable recording. Subsequent staircases were delivered under block of synaptic transmission and after ≥10 minutes of dark adaptation, which sufficed for intrinsic responses to recover from the first staircase (Supplementary Figure 4). These staircases were identical to the first but continued for 3 additional steps to reach 6.3 log R*/rod. These additional steps were excluded from the first staircase because they extended the duration of dark adaptation to beyond the stable lifetime of most recordings (35 °C).

#### Duration-firing relations.

Stimuli for temporal integration experiments consisted either of single pulses (300 s, 30 s, 3 s, 30 ms) or of a set of distributed square pulses (0.1 Hz/0.1 duty cycle or 1 Hz/0.1 duty cycle, given as 30 or 300 pulses across 300 s). Each of these stimuli was calibrated to deliver the same total number of photons per unit area. The standard R* count was 4.85 log R*/rod (equivalent to 6 log photons/μm^2^ at the cornea^25^). Also tested was 5.85 log R*/rod (7 log photons/μm^2^ at the cornea^25^). Stimuli were delivered to each cell in a random order, with intervening dark adaptation periods of ≥5 min. For each trial, the pre-stimulus firing rate was subtracted from the response rate.

### Analysis

#### Statistics.

Non-parametric statistical tests were used unless otherwise noted. Correlations were quantified as Spearman’s rank coefficients. To calculate statistical significance, the two-tailed Mann-Whitney U test was used for unpaired data. Bonferroni corrections were applied when multiple comparisons were made among the same data sets; otherwise, p<0.05 was considered significant. For k means clustering (k=2), Euclidean distance was used and classes were initialized using a random population subset.

#### Estimates of flash threshold for spiking.

Flash intensities tested with <5 trials were omitted. For the remainder, spikes were counted in 100-ms windows, selected from the 500 ms before and after the flash, that contained the maximum and minimum spike counts. Baseline spike rates were subtracted. Three approaches were taken to estimate threshold. The first took the lowest flash intensity in which the pre- and post-flash spike counts appeared different (p = 0.05, one-tailed Wilcoxon signed rank test, Pratt method for handling ties). The second used Receiver Operator Characteristic (ROC) analysis. For each flash intensity, the likelihood of observing *n* spikes after the flash was plotted against that of observing *n* spikes before the flash; varying n gave the ROC curve. The area under each curve was calculated and threshold taken as the lowest flash intensity in which this area was ≥0.60. The third approach was to represent each flash trial as a vector with a value of 1 at each spike time and 0 otherwise, then sum the vectors for every trial of a given flash intensity. This sum was convolved with a Gaussian with a standard deviation of 25 ms (the analysis was robust to reasonable variations of this parameter). Threshold was taken as the lowest flash intensity at which the peak of the convolution was ≥2 standard deviations above the mean, within 0.5 s of flash onset. Thresholds measured by these three methods were comparable. The last was used because it provided the closest match to visual inspection of the data.

#### Comparisons of duration-firing relations.

Permutation tests were used to compare the population duration-firing (D-F) relation in single-pulse, temporal integration experiments. To compare two experimental groups (e.g. 4.85 log R*/rod with and without pupil correction; see **Figure 6**), each cell’s D-F relation was randomly assigned to one of those two groups, keeping the group size consistent with the empirical data set. Single exponentials were then fit to the population D-F relation created from those shuffled data. Trials were excluded when the curve fit failed for either population D-F relation; that is, when either the fit’s X^2^ value exceeded 0.01, or where there was no change in X^2^ after 10 iterations. Each fit yielded a time constant, τ. The difference in τ between the two shuffled population D-F relations was compared to the difference observed empirically. 10,000 shuffles were used. The p value is the fraction of trials in which the difference in τ between shuffled population D-F relations exceeded the empirical value. These p values were 4.85 log R*/rod vs. pupil-corrected 4.85 log R*/rod, 0.0031; 4.85 log R*/rod vs. 4.85 log R*/rod in synaptic antagonists, 0.42; and 4.85 log R*/rod vs. 5.85 log R*/rod, 0.0073.

#### Refitting of behavioral phase shift data from Nelson and Takahashi.

In the original study^3^, behavioral data (phase shift in minutes vs. stimulus intensity in photons/cm^2^/s) were fit to a modified Naka-Rushton equation:

$$R=\frac{R_{min}-R_{max}}{1+{\left(\frac{I}{\sigma }\right)}^{N}}+R_{max}$$

Where R is the response, $R_{min}$ is the minimum response (0 by default), $R_{max}$ is the maximum response, I is the stimulus intensity, σ is the intensity of half-maximal response, and N is a cooperativity factor. To compare these data with cellular duration-firing relations, the raw, per-animal values were refit to this equation; published values of $R_{min}$ and $R_{max}$ were used, while N and σ were free parameters.

#### Software.

Igor Pro (Wavemetrics) was used for curve fitting and most other analyses. Python was used in some cases.

## Supplementary Material

Supplement 1
