# Supplementary material for: Retinal Origins of Circadian Photoregulation's Specialized Dynamic Range and Temporal Integration
Source: bioRxiv. 2025 Dec 30:2025.12.30.695276. Preprint. [Version 1] doi: 10.64898/2025.12.30.695276 (PMC12773001; doi:10.64898/2025.12.30.695276)
Supplement: Supplement 1 [file NIHPP2025.12.30.695276v1-supplement-1.pdf]

bioRxiv preprint doi: https://doi.org/10.64898/2025.12.30.695276; this version posted December 30, 2025. The copyright holder for this preprint (which was not certified by peer review) is the author/funder, who has granted bioRxiv a license to display the preprint in perpetuity. It is made available under aCC-BY 4.0 International license.

	**Baseline Firing (Hz)**	**p**	**Flash Threshold (log_{10} R*/rod/s)**	**p**
**NL$^+$ M4 ipRGC**	9.2 ± 10.9 Hz (4 cells)		-1.70 ± 0.48 (4 cells)	
**CH$^+$ M4 ipRGC**	5.4 ± 8.6 Hz (4 cells)	**vs. NL$^+$ M4:** p = 0.99	-0.88 ± 0.69 (4 cells)	**vs. NL$^+$ M4:** 0.086
**NL$^+$CH$^+$ M4 ipRGC**	**All time points:** 25.1 ± 26.3 Hz (12 cells)<br><br>**>150 min:** 4.5 ± 6.5 Hz (6 cells)	**>150 min vs. CH$^+$ M4:** p = 0.89	-1.04 ± 0.31 (12 cells)	**vs. CH$^+$ M4:** 0.933
		**>150 min vs. NL$^+$ M4:** p = 0.91		***vs. NL$^+$ M4:*** *0.013*
**OFF sustained α-RGC (in NL$^+$ mice)**	15.5 ± 6.8 Hz (6 cells)	**vs. NL$^+$ M4:** p = 0.35	-1.98 ± 0.69 (6 cells)	**vs. NL$^+$ M4:** 0.686
		**vs. CH$^+$ M4:** p = 0.11		**vs. CH$^+$ M4:** p = 0.028
		**vs. NL$^+$ CH$^+$ M4:** p = 0.82		***vs. NL$^+$ CH$^+$ M4:*** *0.008*
**NL$^+$CH$^+$ M1 ipRGC**	2.8 ± 3.3 Hz (32 cells)	**vs. NL$^+$ CH$^+$ M4 (>150 min):** p = 0.043	0.50 ± 0.73 (32 cells)	***vs. NL$^+$ CH$^+$ M4:*** *p < 0.001*

Supplementary Table 1. Minimal desensitization with bioluminescence imaging. Mean ± 1 SD reported for each condition. Statistically significant comparisons (following Bonferroni correction) are italicized.

bioRxiv preprint doi: https://doi.org/10.64898/2025.12.30.695276; this version posted December 30, 2025. The copyright holder for this preprint (which was not certified by peer review) is the author/funder, who has granted bioRxiv a license to display the preprint in perpetuity. It is made available under aCC-BY 4.0 International license.

	**Sensitivity (log pA/R*/rod)**	**p**	**Integration time of flash responses, 440-560 nm (ms)**	**p**
**Wild type**	0.61 ± 0.61 (11 cells)	***vs. Gnat1$^{-/-}$:*** *p = 0.00046*	250 ± 90 (39 flashes, 11 cells)	***vs. Gnat1$^{-/-}$:*** *p < 0.001*
		**vs. Gnat2$^{-/-}$:** p = 0.49		**vs. Gnat2$^{-/-}$:** p = 0.38
		**vs. Opn4$^{Cre/Cre}$:** p = 0.56		**vs. Opn4$^{Cre/Cre}$:** p = 0.027
**Gnat1$^{-/-}$**	-2.23 ± 0.70 (4 cells)	**vs. Gnat2$^{-/-}$:** p = 0.016	90 ± 50 (14 flashes, 5 cells)	***vs. Gnat2$^{-/-}$:*** *p < 0.001*
		vs. Opn4$^{Cre/Cre}$: *p = 0.00046*		***vs. Opn4$^{Cre/Cre}$:*** *p < 0.001*
**Gnat2$^{-/-}$**	0.85 ± 0.62 (4 cells)	**vs. Opn4$^{Cre/Cre}$:** p = 0.95	250 ± 80 (14 flashes, 4 cells)	**vs. Opn4$^{Cre/Cre}$:** p = 0.018
**Opn4$^{Cre/Cre}$**	0.80 ± 0.63 (11 cells)		200 ± 30 (11 flashes, 11 cells)	

Supplementary Table 2. Sensitivity and kinetics indicate rod origins of threshold responses in M1 ipRGCs. Statistically significant comparisons (following Bonferroni correction) are italicized.

bioRxiv preprint doi: https://doi.org/10.64898/2025.12.30.695276; this version posted December 30, 2025. The copyright holder for this preprint (which was not certified by peer review) is the author/funder, who has granted bioRxiv a license to display the preprint in perpetuity. It is made available under aCC-BY 4.0 International license.

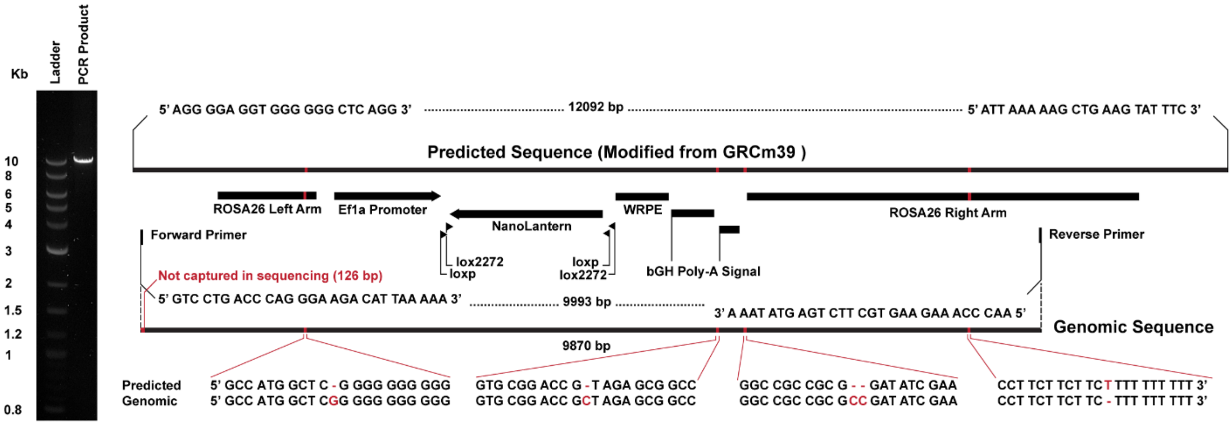

Supplementary Figure 1. Validation of knock-in at the *ROSA26* locus. *Left*, PCR product from the *ROSA26* locus of putative *ROSA26*$^{nano\text{-}lantern/nano\text{-}lantern}$ animals. *Right,* Alignment of the genomic sequence, derived from sequencing the purified PCR amplicon, against the mouse reference genome (GRCm39) modified to include the nano-lantern knock-in sequence. Solid horizontal lines indicate sequence alignments (black) and misalignments (red; not to scale). Sequence annotations, PCR primers and their annealing loci, as well as a 126-bp sequence not captured in sequencing are depicted in between the predicted and genomic sequences.

bioRxiv preprint doi: https://doi.org/10.64898/2025.12.30.695276; this version posted December 30, 2025. The copyright holder for this preprint (which was not certified by peer review) is the author/funder, who has granted bioRxiv a license to display the preprint in perpetuity. It is made available under aCC-BY 4.0 International license.

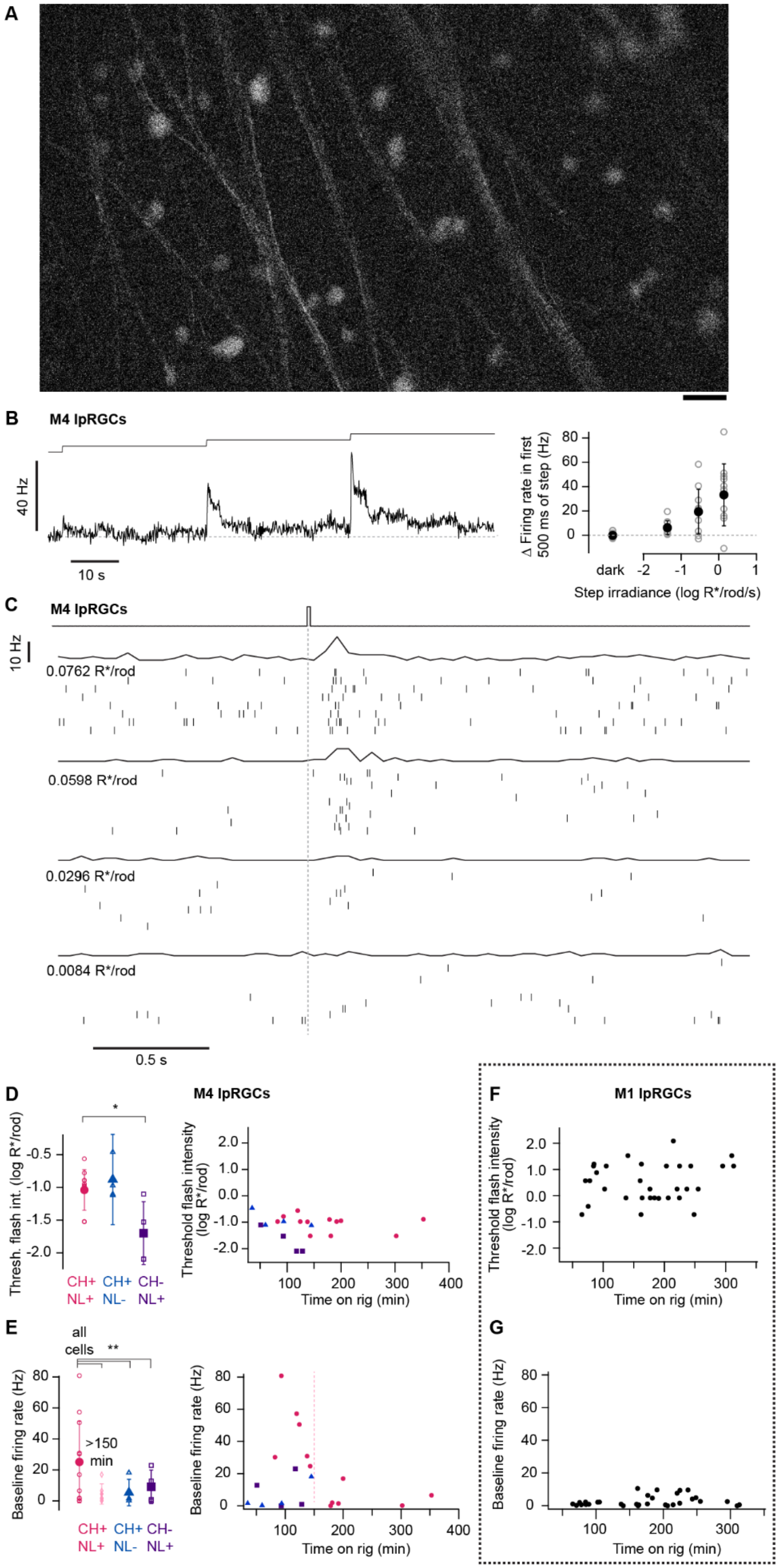

bioRxiv preprint doi: https://doi.org/10.64898/2025.12.30.695276; this version posted December 30, 2025. The copyright holder for this preprint (which was not certified by peer review) is the author/funder, who has granted bioRxiv a license to display the preprint in perpetuity. It is made available under aCC-BY 4.0 International license.

Supplementary Figure 2. Bioluminescence imaging preserves sensitivity.

A. Example of bioluminescence from an Opn4$^{Cre/+}$;ROSA26$^{NL/+}$ retina that was exposed to coelenterazine h (25 µM CH, 5 min) and then imaged (10-s exposure, CMOS camera). 20-µm scale bar.

B. *Left,* M4 ipRGCs were identified by bioluminescence and their light responses (**Methods**). Spikes were recorded in loose-patch mode at 35 ºC. A staircase was given from -1.4 to 0.1 log R*/rod. The average firing rate of 11 cells is shown (100-ms bins). Baseline firing in darkness is indicated (dashed line). The stimulus fully covered the dendritic arbor. *Right,* Transient increases in firing rate with increasing step intensity for the sample. Filled circles are the average ±1 SD.

C. M4s were identified by bioluminescence and recorded as in **B**. Flashes of different intensities (15-30 ms, stimulus monitor at top, 410 nm) were given to estimate threshold. Spike firing rates (100-ms bins) are shown above sample rasters. The stimulus fully covered the dendritic arbor.

D. *Left,* Summary statistics of flash threshold (the lowest flash intensity producing detectable spike modulation) of M4s (see **Supplementary Table 1** for r and p values). Experimental cells were bioluminescent; they expressed nano-lantern (NL) and were exposed to CH (CH^{+} NL^{+}, circles). Control cells were exposed to CH but did not express NL (CH^{+} NL^{-}, triangles), or expressed NL but were not exposed to CH (CH^{-} NL^{+}, squares). The difference between CH+ and CH- may be due to luminescence from CH oxidation[52]. *Right,* Flash thresholds plotted against time on the rig (which initiates CH washout and thus bioluminescence decay). M4 thresholds were stable over time.Supplementary Spikes were recorded in loose-patch mode at 35 ºC.

E. As in **D**, but for baseline firing rate rather than threshold flash intensity. M4s in CH^{+} NL^{+} retinas showed elevated baseline firing initially (<150 min on the rig) but those recorded >150 min after CH washout (*right,* diamonds) had baseline firing rates that were indistinguishable from control M4s (see **Supplementary Table 1** for r and p values).

F. As in the right panel of **D**, but for NL-expressing M1 ipRGCs (transduced retrogradely from the hypothalamus). M1 thresholds show no dependence on time since washout; their thresholds are also higher than those of M4s (see **Supplementary Table 1** for r and p values).

G. As in **F**, but for baseline firing rather than threshold flash intensity. M1 firing rates in darkness showed no dependence on time since CH washout (see **Supplementary Table 1** for r and p values).

bioRxiv preprint doi: https://doi.org/10.64898/2025.12.30.695276; this version posted December 30, 2025. The copyright holder for this preprint (which was not certified by peer review) is the author/funder, who has granted bioRxiv a license to display the preprint in perpetuity. It is made available under aCC-BY 4.0 International license.

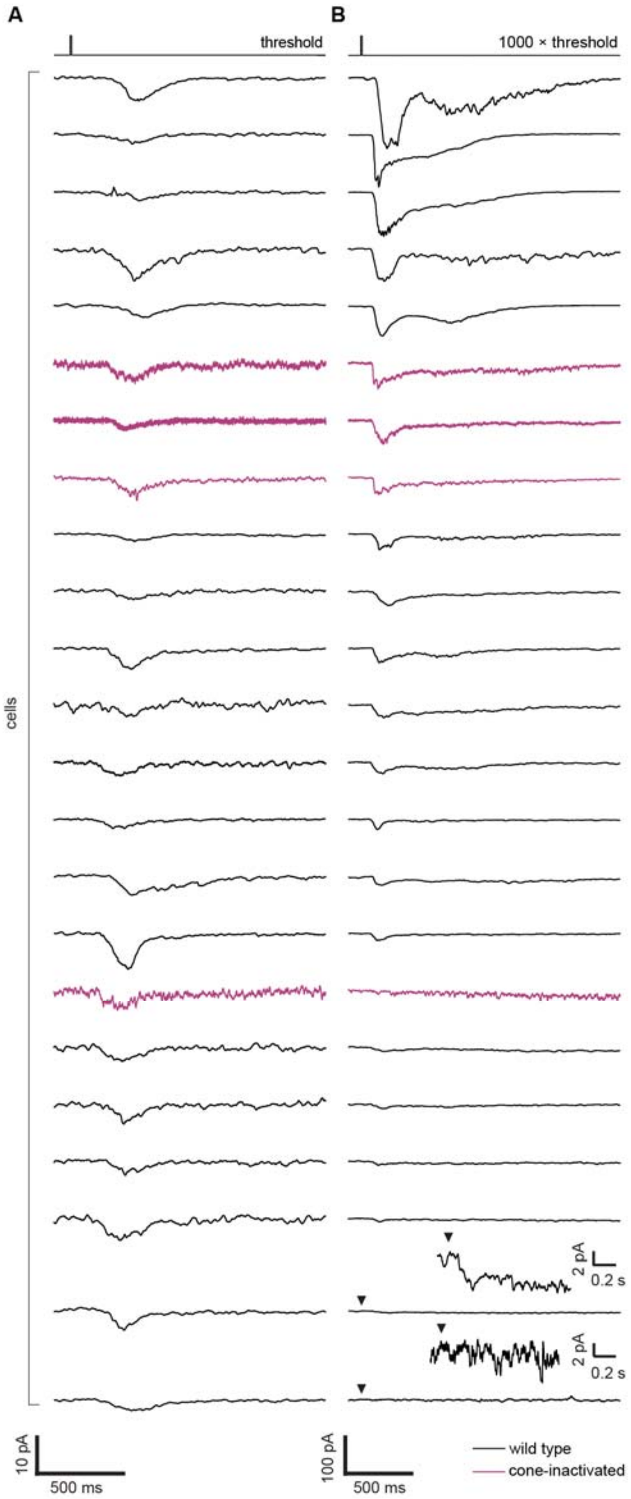

Supplementary Figure 3. High flash intensities reveal diversity of synaptic responses in M1 ipRGCs.

A. Near-threshold flash responses of 23 M1s, recorded in voltage clamp at -80 mV. Stimulus flashes (top trace showing square pulse) were 20-30 ms and 500 nm, and their intensity varied

bioRxiv preprint doi: https://doi.org/10.64898/2025.12.30.695276; this version posted December 30, 2025. The copyright holder for this preprint (which was not certified by peer review) is the author/funder, who has granted bioRxiv a license to display the preprint in perpetuity. It is made available under aCC-BY 4.0 International license.

from 0.034 – 0.42 log R*/rod; response amplitudes varied from 2-10 pA. Cells are from wild-type (black) or *Gnat2*$^{-/-}$ (magenta) retinas. The latter have inactivated cones but normal rod and melanopsin responses. Traces are averages of 6-40 sweeps. Recordings were made at 23 ºC for additional stability.

B. Responses from these same 23 cells to flashes calibrated to 1000× each cell's approximate threshold. Traces are averages of 1-5 sweeps. Cells in **A** and **B** are ordered by the strength of their response to this flash; response amplitudes varied from undetectable (bottom row) to ~200 pA (top row). Insets show the two smallest responses (bottom two rows) on an expanded current base (arrowheads mark the onset of the flash).

bioRxiv preprint doi: https://doi.org/10.64898/2025.12.30.695276; this version posted December 30, 2025. The copyright holder for this preprint (which was not certified by peer review) is the author/funder, who has granted bioRxiv a license to display the preprint in perpetuity. It is made available under aCC-BY 4.0 International license.

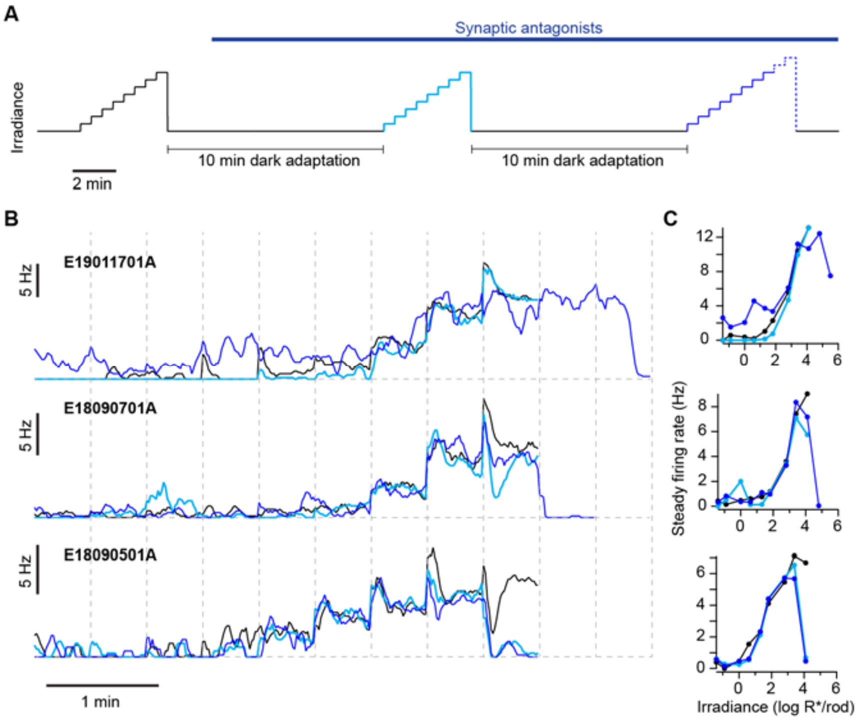

Supplementary Figure 4. Recovery of M1 intrinsic photosensitivity following intensity staircases

A. Cells were presented with an intensity staircase (black; 8 steps, 30-s each, -0.9 to 4.1 log R*/rod/s, 460-nm light). During the subsequent 10 minutes of dark adaptation, synaptic antagonists were added to isolate the intrinsic light responses (**Methods**). Antagonists remained throughout the rest of the recording. The same staircase was presented a second time (cyan) and, after another 10-min dark adaptation, a third time (blue). Occasionally, the third staircase included 1-2 additional intensity steps, which reached a maximum of 5.5 log R*/rod/s (dashed line). Recordings were performed in loose-patch mode at 35° C.

B. Overlaid firing rates from the three staircases (black, cyan, and blue traces) for three example cells (top, middle, and bottom rows; 5-s moving average). The last two epochs correspond to additional intensity steps (see **A**).

C. Intensity-firing relations for the data in **B**.

bioRxiv preprint doi: https://doi.org/10.64898/2025.12.30.695276; this version posted December 30, 2025. The copyright holder for this preprint (which was not certified by peer review) is the author/funder, who has granted bioRxiv a license to display the preprint in perpetuity. It is made available under aCC-BY 4.0 International license.

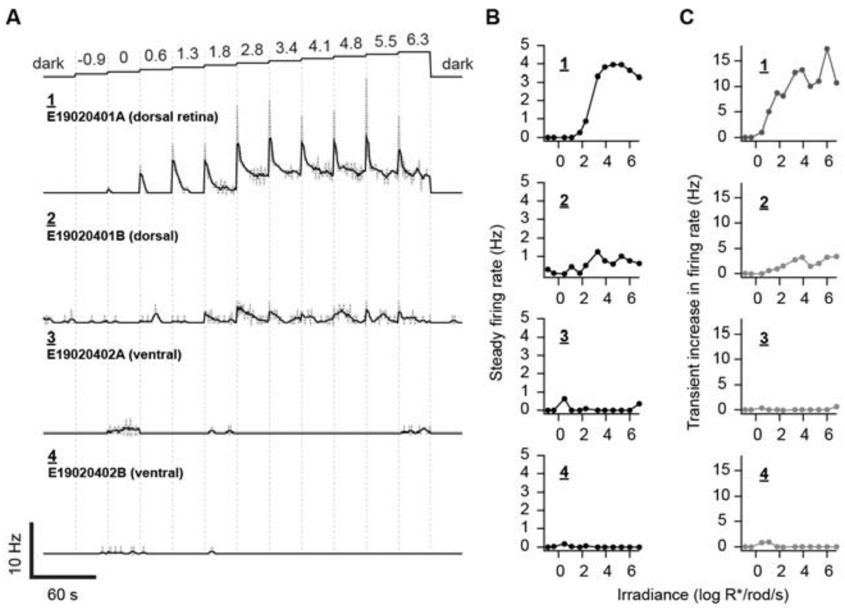

Supplementary Figure 5. M1 intensity-firing relations without melanopsin

A. The extended staircase stimulus (see **Figure 2**; all intensity values are in log R*/rod/s) was delivered to 4 Opn$^{Cre/Cre}$ M1s (rows 1-4). Cells 1 and 2 (**E19020401A/B**) were recorded simultaneously in the dorsal retina, and cells 3 and 4 (**E19020402A/B**) were recorded simultaneously in the ventral half of the same retina. Loose-patch recordings, no synaptic antagonists, 35° C.

B-C. Intensity-firing relations for the four cells in **A**, reflecting steady firing during the last 10 s of each step (**B**) or transient firing during the first 500 ms of each step (**C**). Cells 3 and 4 have virtually no light responses; cell 2 has a modest response with little intensity encoding, and cell 1 is able to encode intensity across several log units.

bioRxiv preprint doi: https://doi.org/10.64898/2025.12.30.695276; this version posted December 30, 2025. The copyright holder for this preprint (which was not certified by peer review) is the author/funder, who has granted bioRxiv a license to display the preprint in perpetuity. It is made available under aCC-BY 4.0 International license.

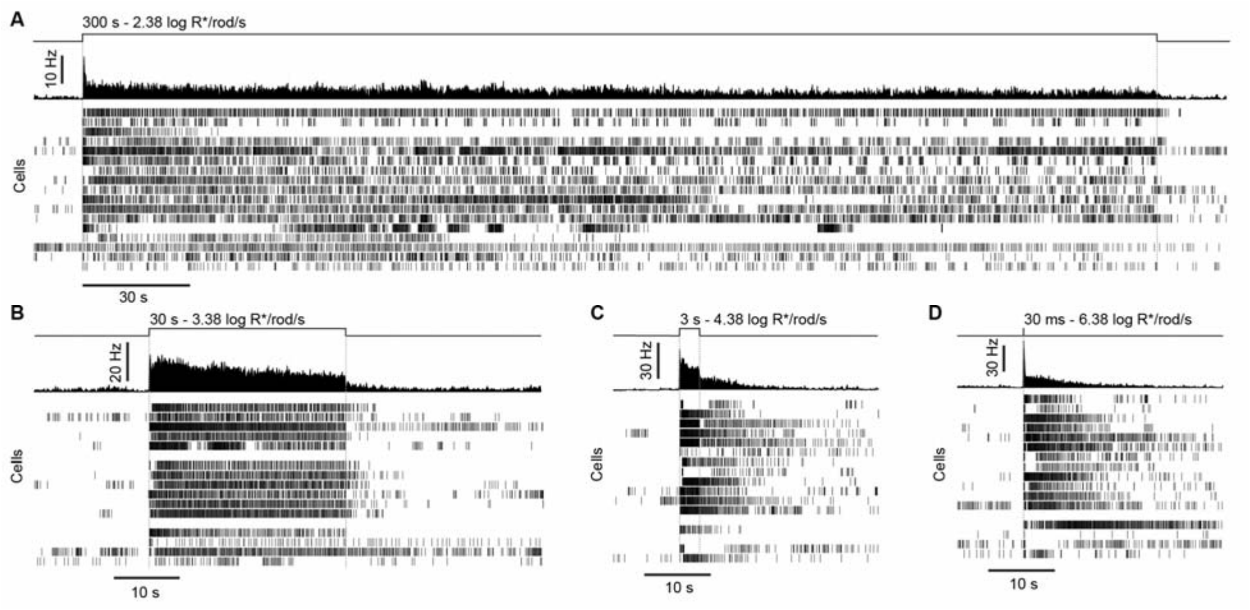

Supplementary Figure 6. Individual M1 responses to single-pulse temporal integration stimuli.

A-**D**. Average spike rates (top) and individual spike rasters for all M1 responses to the 300 s (**A**, 17 cells), 30 s (**B**, 15), 3 s (**C**, 15), and 30 ms (**D**, 16) stimuli. Each stimulus delivered a total of 4.85 log R*/rod. Stimulus monitors at top. Stimuli were delivered in random orders across cells, with >300 s of dark adaptation between presentations. Each row is one trial from one cell. Not all cells received all four stimulus durations; blank rows are these missing data. Loose-patch recording, 35°C, no synaptic antagonists.

bioRxiv preprint doi: https://doi.org/10.64898/2025.12.30.695276; this version posted December 30, 2025. The copyright holder for this preprint (which was not certified by peer review) is the author/funder, who has granted bioRxiv a license to display the preprint in perpetuity. It is made available under aCC-BY 4.0 International license.

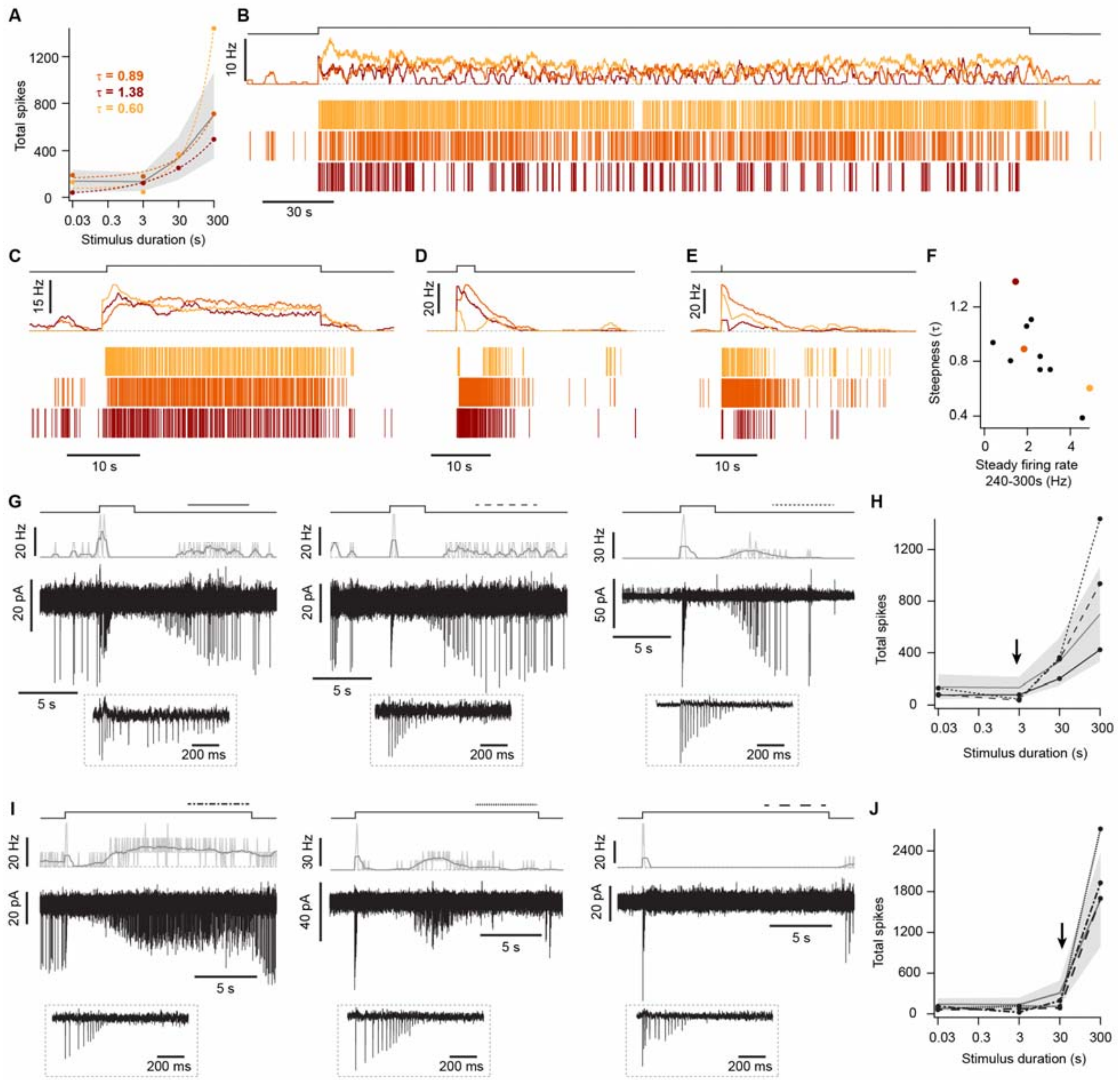

Supplementary Figure 7. Sources of variability in temporal integration across M1 ipRGCs

A. Duration-Firing (D-F) relations for three example M1s from the data set shown in **Figure 3D** and **Supplementary Figure 6**. Stimuli delivered a total of 4.85 log R*/rod over different durations, and produced varying spike numbers across the example cells. Individual data points (circles) are colored by cell identity and overlaid with single exponential fits (dashed lines, with τ reported for each). The black line and grey shading are mean ± SD. Loose-patch recording, 35 °C, no synaptic antagonists.

B-E. Spike rates (above) and rasters (below) for the three example cells in **A** when given the 300 s (**B**), 30 s (**C**), 3 s (**D**), or 30 ms (**E**) stimulus. Histograms are shown as a 0.5-s moving average of 0.1-s bins. Stimulus monitors at top.

F. The τ of all D-F relations in the data set of **Figure 3E** (11 of 17 cells whose relations could be fit with a single exponential). Steeper D-F relations, which have less precise temporal

bioRxiv preprint doi: https://doi.org/10.64898/2025.12.30.695276; this version posted December 30, 2025. The copyright holder for this preprint (which was not certified by peer review) is the author/funder, who has granted bioRxiv a license to display the preprint in perpetuity. It is made available under aCC-BY 4.0 International license.

integration, have lower τ values. τ was correlated with the steady firing rate (measured in the last 60 s of the 300-s stimulus; Spearman's r = -0.68, p = 0.03).

G. 3 example M1s for which the 3-s, single-pulse stimulus (4.85 log R*/rod) produces spike silencing. Shown are stimulus monitors (shaded), spike rates (light gray: 0.1-s bins, dark gray: 0.5-s moving average), and raw traces. Insets show the first 1 s after stimulus onset. Spike amplitudes dwindling into noise, then recovering after stimulus cessation, is a signature of depolarization block[27]. Thus, depolarization block shapes temporal integration of M1s.

H. D-F relations for the 3 cells in **G-I** (black lines with markers), with the population mean D-F relation for 4.85 log R*/rod (solid gray line, shading is ±1 SD) given for comparison. The arrow marks the stimulus that produced depolarization block in these cells. Note that all 3 have below-average responses to this stimulus.

I. 3 example M1s given a 30-s stimulus delivering 5.85 log R*/rod (same photon flux density but 10-fold longer duration than for the cells in **G**; see Figure **3E**). Cells showed varying degrees of depolarization block: blocking transiently and resuming firing during the stimulus (*left*), transiently resuming firing mid-stimulus and blocking again (*center*), and staying in block throughout the stimulus and only resuming firing in darkness (*right*).

J. D-F relations for the 3 cells in **I**, compared to the population mean D-F relation for 5.85 log R*/rod (solid green line, shading is ±1 SD). The arrow marks the stimulus that produced depolarization block in these cells. Note that all 3 have below-average responses to this stimulus.
